# Anti-SARS-Cov-2 IgA Response in Tears of COVID-19 Patients

**DOI:** 10.3390/biology9110374

**Published:** 2020-11-03

**Authors:** Elisabetta Caselli, Irene Soffritti, Giuseppe Lamberti, Maria D’Accolti, Filippo Franco, Davide Demaria, Marco Contoli, Angela Passaro, Carlo Contini, Paolo Perri

**Affiliations:** 1 Department of Chemical and Pharmaceutical Sciences and LTTA, Section of Microbiology, University of Ferrara, 44121 Ferrara, Italy; elisabetta.caselli@unife.it (E.C.); irene.soffritti@unife.it (I.S.); maria.daccolti@unife.it (M.D.); 2 Department of Biomedical and Surgical-Specialized Sciences, Ophthalmology Unit, University of Ferrara, 44121 Ferrara, Italy; g.lamberti@ausl.fe.it (G.L.); filippo.e.franco@tiscali.it (F.F.); davide.demaria@unife.it (D.D.); 3 Department of Morphology, Surgery and Experimental Medicine, University of Ferrara, 44121 Ferrara, Italy; marco.contoli@unife.it (M.C.); angelina.passaro@unife.it (A.P.); paolo.perri@unife.it (P.P.); 4 Department of Medical Sciences, Infectious Diseases Unit, University of Ferrara, 44121 Ferrara, Italy

**Keywords:** SAR-CoV-2, IgA, eye, COVID-19

## Abstract

**Simple Summary:**

SARS-CoV-2 can enter the body via the eye but the local antiviral response is still poorly known, we thus analyzed the presence of mucosal antibodies in the tears of COVID-19 patients. The results show that 35.7% of COVID-19 subjects have specific antiviral secretory antibodies in the eye. Their detection may be extremely helpful in clarifying, at this level, virus pathology and epidemiology.

**Abstract:**

The pandemic virus SARS-CoV-2 has been reported to be able to enter the body via the eye conjunctiva, but the presence of antiviral response in the eye remains poorly known. Our study was thus aimed to analyze the presence of secretory mucosal anti-SARS-CoV-2 type A immunoglobulins (IgA) in the conjunctival fluid of COVID-19 patients. The tears of 28 COVID-19 patients and 20 uninfected controls were collected by the Schirmer test and analyzed by a specific ELISA assay detecting anti-spike (S1) virus protein IgA. The results showed that 35.7% of COVID-19 subjects have specific antiviral IgA at the ocular level, persisting till 48 days post disease onset. Most of the IgA positive subjects presented mild symptoms. The collected data indicate a prolonged persistence of anti-SARS-CoV-2 IgA at the eye level and suggest that IgA detection may be extremely helpful in clarifying virus pathology and epidemiology.

## 1. Introduction

The infection by the severe acute respiratory syndrome coronavirus 2 (SARS-CoV-2) is the etiological cause of the coronavirus disease 2019 (COVID-19), which is rapidly spreading in the human population [1]. Originally identified in Wuhan, China, in December 2019, the World Health Organization (WHO) considered it a pandemic at the beginning of 2020 and consequently provided a guide for protection, diagnosis, and treatment protocols according to the rising emergency [2,3].

To date, the infection has killed more than 1 million people, with more than 44 million cases and 1.17 million deaths in the world at the end of October 2020, including Europe, in which currently there have been over 265,000 deaths. The number of infected cases is steadily increasing especially in the USA, South America (Brazil), South East Asia, and Africa. The COVID-19 origin is still discussed, although the initial cases have been associated with the Huanan South China Seafood Market. However, there is no absolute evidence so far that the origin of SARS-CoV-2 was from the seafood market [1]. SARS-CoV-2 is a beta-coronavirus likely of zoonotic origin, as for the previous severe acute syndrome coronavirus (SARS-CoV-1) and Middle East respiratory syndrome coronavirus (MERS-CoV) [4].

This new pandemic virus has been classified as a novel Betacoronavirus, belonging to the Sarbecovirus subgenus of the *Coronaviridae* family. This virus has never previously been identified in humans and information coming from the scientific world is constantly evolving. The genome sequence of SARS-CoV-2 is about 89% identical to bat SARS-like-CoV and 82% identical to human SARS-CoV-1 [5]. Phylogenetic analysis of novel SARS-CoV-2 has shown that it is a product of recombination with previously identified bat coronaviruses. However, significantly, SARS-CoV-2 is only closely related to the specific bat SARS-like coronavirus isolated from *Rhinolophus sinicus* in 2015 in China (MG772934.1). Further structural analysis of two important viral proteins, the nucleocapsid (N protein) involved in virion assembly and the S-protein-like nucleoprotein (S has confirmed a significant similarity between the new coronavirus and the bat-like SARS-CoV while underlining its difference from the SARS-CoV-2 [6,7]. If these proteins undergo mutations, the result could lead to a greater ability to infect than the bat-like SARS-CoV, but a lower pathogenicity than the SARS-CoV-1. This may explain its initial lower severity when compared to the SARS epidemic [8].

SARS-CoV-2 is reported as using the same cell entry receptor as SARS-CoV [9], the angiotensin-converting enzyme 2 (ACE2), to infect humans, so clinical similarities between the two viruses are expected, particularly in severe cases. ACE2 is a crucial enzyme in the Renin-Angiotensin system and a target for the treatment of hypertension [10]. SARS-CoV-2 recognizes human ACE2 receptor more efficiently than SARS-CoV and this may increase the ability of SARS-CoV-2 to be transmitted from person to person, resulting in a more efficient spread among subjects [9]. The abundant expression of the ACE2 receptor in type II alveolar cells promotes a rapid viral multiplication with the subsequent destruction of the local alveolar wall. This process results in rapidly progressive and widespread alveolar damage with the occurrence of concomitant cytokine/chemokine cascades [11].

ACE2 receptor has a wide distribution in the organs and tissues of the human body, including intestine, heart, kidney, and endothelium, which could explain the multi-organ dysfunction observed in patients.

The pandemic diffusion of SARS-CoV-2 is likely related to its ability to replicate efficiently in the human upper respiratory tract, together with the presence of a high multitude of asymptomatic and poorly symptomatic subjects, which facilitates virus human-to-human transmission by aerosol droplets [1] and rapid spread of viral contagion. In symptomatic patients, SARS-CoV-2 infection can cause a mild flu-like disease to a critical illness with multifaceted aspects which can be lethal especially in the elderly [1,12].

Since the death of an ophthalmologist in Wuhan in February 2020, the possibility that SARS-CoV-2, responsible for COVID-19, can infect the eye and be transmitted through ocular fluids has been investigated [1]. Other respiratory viruses can in fact cause ocular infections and establish respiratory infections following ocular exposure. This was first described for the RNA respiratory syncitial virus (RSV), showing a clear eye-to lung route in the mouse animal model [13]. The mucosal surface of the eye is in fact exposed to infectious aerosols and several viruses can establish a respiratory infection following ocular exposure, including both DNA and RNA viruses, such as adenovirus, influenza virus, rhinovirus, human metapneumovirus, and human coronavirus [13,14]. Ocular involvement was also observed in non-respiratory diseases, like the acquired immunodeficiency syndrome (AIDS), where the incidence of ocular manifestations was inversely proportional to the use of highly active antiretroviral therapy (HAART) [15].

Consistent with the previous studies on the human coronaviruses SARS-CoV-1 and MERS, the recent research on SARS-CoV-2 indicates that, although the virus is spread primarily by respiratory droplets, the conjunctiva can be considered as a potential way for both virus entry into the body and as a source of infection [16]. Indeed, there is uncertainty whether conjunctival epithelial cells express sufficient amounts of ACE2 receptor to allow optimal cell entry of the virus in conjunctival cells and efficient propagation of the virus in the conjunctiva, as immunohistochemical staining studies using two different monoclonal antibodies found no expression of ACE2 in conjunctival tissue [17]. Nevertheless, ACE2 and its receptor subtypes have been found in the human retina [18] and conjunctival cells, as well as in mouse cornea [19], suggesting potential virus entry and spread through this route [20]. Furthermore, both ACE2 and transmembrane protease serine 2 (TMPRSS2) cofactor are highly expressed.

Many health authorities’ records report the infection of ophthalmologists worldwide, possibly linked to their clinical activity. However, it has not been clarified whether the infection was due to eye route, oral droplets, aerosol-breath, or others [21]. The close proximity of ophthalmologists with infected patients increases in fact all these possible routes, although contamination by conjunctiva in the maneuvers needed for conjunctival evaluation as well as direct contact with the infected conjunctiva may lead to infection transmission [22,23].

Based on these observations, research has so far mainly focused on finding evidence for the presence of SARS-Cov-2 in ocular secretions, to assess the potential virus spread via ocular fluids [16,24]. Most studies searched virus RNA in ocular swabs by RT-PCR assays, showing its presence in a variable and generally low proportion of analyzed conjunctival samples. Overall, the frequency of SARS-CoV-2 detection in ocular samples is very low, ranging from 0 to about 7%, and the WHO-China Joint Mission on COVID-19 estimated conjunctival congestion in 0.8% of infected individuals, based on a study on 55,924 laboratory-confirmed cases [25]. So far, very few studies tried, besides RNA detection, parallel virus isolation from eye samples, by culturing the specimens on Vero_E6 cells, and only one positive case was reported in a patient with conjunctivitis in Italy [26].

However, despite the high number of studies regarding the presence of infectious SARS-CoV-2 or its genomic material at the eye level, there is a lack of information about the local immune response against the virus, which could instead be highly informative. Notably, the eye is an immunoprivileged site, where soluble and cellular factors can contribute to the development of tolerogenic activity in response to the presence of foreign antigens [27]. Consequently, it would be important to investigate the presence of an immune response within the eye, also evaluating its possible correlation with the disease clinical severity and duration, as well as with patient characteristics, such as age and comorbidities.

The present study was thus aimed to evidence the presence of secretory IgA specifically directed against the SARS-CoV-2 virus in the eye of COVID-19 patients.

## 2. Materials and Methods

### 2.1. Patients and Samples

Twenty-eight COVID-19 patients were recruited at the University-Hospital of Ferrara (Italy) after obtaining informed consent and approval by the local Ethics Committee (Trial registration: isrctn.org identifier ISRCTN17301812). All COVID-19 subjects were positive for SARS-CoV-2 detection by polymerase chain reaction after reverse transcription (RT-PCR) performed on nasopharyngeal/oropharyngeal swabs (NPS/OPS).

Twenty healthy subjects were also enrolled as controls, including 10 persons belonging to the sanitary staff and 10 patients referred for ocular cataract surgery. All control subjects were negative for both SARS-CoV-2 presence in NPS/OPS and anti-SARS-CoV-2 serology.

Tears were sampled using a Schirmer test strip from both eyes, as previously reported [28]. The obtained strips were immediately placed in sterile microtubes containing 0.4 mL sterile PBS, which were refrigerated and transported to the laboratory within 2 h. Collected samples were then vortexed and centrifuged to collect eluates, which were then frozen at −80 °C until use.

### 2.2. IgA Detection

The presence of anti-SARS-CoV-2 IgA in the tears obtained from COVID-19 patients and uninfected controls was evaluated by a CE-IVD ELISA assay designed to detect IgA directed against the virus S1 protein (Euroimmun, Lubeck, Germany). The used test was previously reported to have high specificity and sensitivity for the detection of IgA in serum/plasma samples (>95%) [29]. To optimize the manufacturer’s protocol for tear analysis, tear samples were tested at serial dilution in PBS before sample analysis. Based on the results, tear samples were diluted 1:5 in saline, allowing optimal detection of IgA and differentiation between positive samples and controls. Following the manufacturer’s instructions, tear samples’ positivity was expressed as the ratio (R) between the OD_450nm_ values detected in tested samples and that obtained in the calibrator sample provided by the manufacturer, representing the threshold of positivity. Each sample was assessed in triplicate. Samples were considered negative when R values were <0.8, weakly positive with R values comprised between 0.8–1.1, and strongly positive with R ≥ 1.1.

### 2.3. Statistics

Individual parameters of the subjects enrolled in the study groups were expressed as mean values and corresponding standard deviation or value range. The non-parametric Mann–Whitney test was used for the comparison between patient and control groups. The correlation between presence/amount of ocular IgA and patients’ variables was calculated by linear regression. Parameters evaluated in the correlation analysis included age, gender, disease severity, and ocular symptoms. *p* < 0.05 were considered statistically significant.

## 3. Results

Twenty-eight COVID-19 patients were recruited at the University-Hospital of Ferrara (Italy) after obtaining informed consent and approval by the local Ethics Committee.

The recruited COVID-19 patients had a mean age of 66 ± 18 years (range 19–89) and included 14 males and 14 females (Table 1). All of them tested positive for SARS-CoV-2 by RT-PCR analysis of nasopharyngeal/oropharyngeal swabs (NPS/OPS). Recruited patients were all symptomatic at hospitalization, showing mild-to-severe clinical manifestations, including fever, respiratory and/or gastrointestinal symptoms. Only one patient presented, ocular symptoms, consisting of mild eye redness.

The control group consisted of SARS-CoV-2 negative subjects including 10 volunteers recruited among the sanitary personnel and 10 patients referred for ocular cataract surgery. Control subjects included two males and 18 females, with a mean age of 64 ± 11 years (range 48–85).

Subjects’ conjunctival fluid was collected by the Schirmer test strip from both eyes. In the COVID-19 group, tear samples were collected from 1 to 78 days after the hospitalization and NPS/OPS test.

The eluates obtained from both COVID-19 and control groups were tested for the presence of anti-SARS-CoV-2 IgA by using a CE-IVD ELISA assay specifically evidencing the antibodies directed against the virus S1 protein, after optimizing and validating the protocol for analysis of conjunctival fluid. Following the manufacturer’s instructions, the level of positivity was calculated as the ratio (R) between the absorbance values of samples and calibrator, at OD_450nm_ (R < 0.8, negative; 0.8 ≤ R < 1.1, weakly positive; R ≥ 1.1, strongly positive).

**Figure 1:**
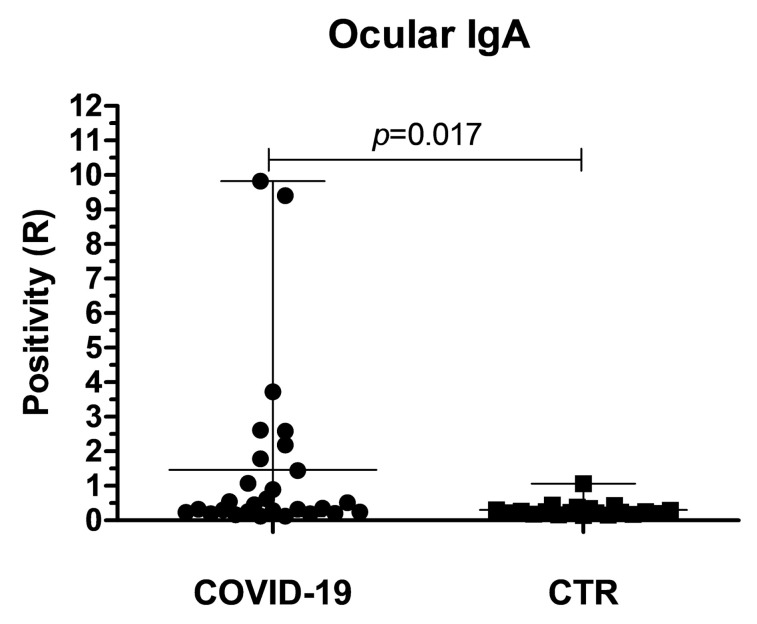
Presence of anti-SARS-CoV-2 specific IgA in the tears of COVID-19 patients and control subjects. Plotted values were calculated following the manufacturer’s instructions and expressed as the ratio (R) between the OD_450nm_ values detected in tested samples and calibrator. Each sample was assessed in triplicate. Mean and range values of both groups are also indicated, corresponding to 1.464 ± 0.46 in COVID-19 and 0.301 ± 0.04 in the control group, respectively. The *p* value was calculated by the Mann–Whitney non parametric test.

The results evidenced the presence of ocular anti-SARS-CoV-2 IgA in 10/28 (35.7%) of the enrolled COVID-19 patients, with variable concentrations, ranging from R = 0.896 ± 0.01 (weakly positive) to R = 9.822 ± 0.13 (strongly positive) (Figure 1). Eight samples resulted in being strongly positive (R > 1.1) whereas only two showed a weak positivity (with R = 0.896 and 1.076, respectively). The data evidenced the presence of two populations among the COVID-19 patients, one of which displaying clearly detectable IgA response in the eye conjunctiva.

By contrast, no control samples were found to be positive for the presence of anti-SARS-CoV-2 IgA, except for one sample derived from a subject belonging to the sanitary staff subgroup, which resulted in weak positivity for IgA presence (R = 1.068 ± 0.32) (Figure 1).

The mean R values detected in COVID-19 and control groups were 1.464 ± 0.46 and 0.301 ± 0.04, respectively and the difference between the study groups mean R values resulted statistically significant by the non-parametric Mann–Whitney test (*p* = 0.017).

**Figure 2:**
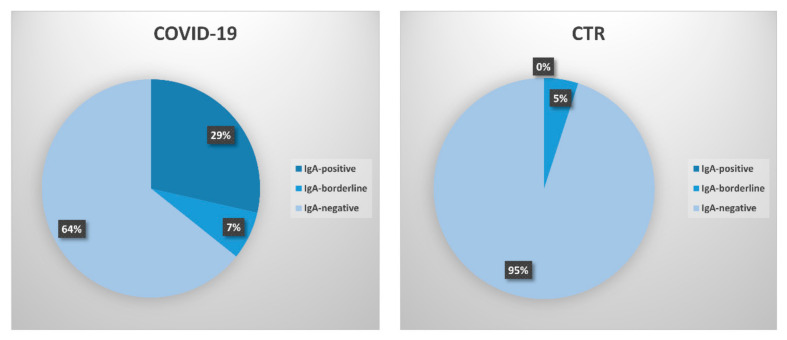
Percentage of positivity in COVID-19 patients and control subjects. Samples are subdivided based on their R value, as calculated following the manufacturer’s instructions. IgA-positive samples are defined as strongly positive samples with R ≥ 1.1; IgA-borderline samples are defined as samples which were weakly positive (0.8 ≤ R < 1.1); IgA-negative samples are defined as those having R < 0.8.

As well, the positivity percentages of subjects in the COVID-19 and control groups were highly statistically significant (*p* < 0.001) (Figure 2). No frankly positive samples were detected in the control group.

Based on the seeming subdivision of the COVID-19 group into two sub-populations responding differently at the mucosal level, we analyzed the possible correlation of the IgA eye response with clinical parameters of the patients. However, no significant correlation was observed with any of the analyzed parameters by linear regression and correlation analyses. The presence and concentration of ocular IgAs in the COVID-19 group appeared in fact seemingly unrelated to all the examined factors, including age, gender, lymphocyte number, and presence of ocular symptoms (Table 2). No significant correlation was observed between IgA presence/concentration and disease stage and severity. No significant correlation was observed between IgA presence/concentration and disease severity; however, pneumonia/acute respiratory distress syndrome (ARDS) was present in only 3/10 (30%) subjects, whereas the remaining patients displayed mild or no respiratory symptoms. Of note, five cases with undetectable IgA response presented severe symptoms including cardiac failure, bowel obstruction, and gastric hemorrhage. Besides, among the IgA-negative COVID-19 subjects, six patients had constitutive immune deficiencies that might have hampered the development of an anti-SARS-CoV-2 IgA response, including leukemia, lymphomas, very advanced age, and chemotherapy treatment due to concomitant cancer.

Notably, the detection of a strong IgA response up to 48 days after the first positive NPS/OPS suggested a long persistence of the anti-SARS-CoV-2 IgA response in the eye.

## 4. Discussion

The COVID-19 pandemic has presented major challenges to ophthalmologists. Like many other respiratory viruses, the SARS-CoV-2 virus could also be transmitted by contact with the eye surface, as demonstrated by the detection of viral RNA in conjunctival specimens while ocular manifestations can be the first presenting symptoms of COVID 19 infection [22]. Based on this, many studies have been performed on the search of infectious virus in the eye [16,24,25,26,30]. Collected data suggest that the virus may enter the body and spread through this route, as SARS-CoV-2 genomic RNA has been detected at the eye level, although in a low percentage of COVID-19 patients [25].

The possibility that the conjunctiva may be a site of virus replication has been indicated in several reports, in cross-sectional as well as monocentric studies or case reports [16,20,22,24,26,31,32,33,34], posing an important concern for infection prevention in healthcare workers and the general population, and highlighting the need for eye protection in addition to respiratory protection. Despite the lack of frequent isolation of infectious SARS-CoV-2 from the conjunctiva [26], the eye is thus considered a potential portal of entry and spread for the virus. In addition, coronaviruses in general are recognized as possible causal agents for uveitis, choroiditis, and blood- retinal barrier breakdown in animal models [35], suggesting that SARS-CoV-2 may be associated with intraocular inflammation and other ocular symptoms. Overall, the most common ocular manifestation of SARS-CoV-2 infection is unilateral or bilateral conjunctivitis or conjunctivitis-like signs and symptoms, which is similar to that observed in SARS-CoV-1 and HCoV-NL63 infections [36]. Other ocular manifestations include photophobia, redness, ocular secretions, chemosis, itching, foreign body sensation, dry eyes, follicular conjunctivitis, and episcleritis, all symptoms which may simulate other ocular diseases [37]. There has been only one study showing retinal changes in COVID-19 patients [38].

By contrast, the development of a local immune response against the virus in the conjunctival fluid and its eventual clinical meaning is still unknown.

Our study thus aimed to evaluate the presence of secretory IgA in the tears of COVID-19 patients with different degrees of symptoms and disease stage. The tears were collected by the non-invasive Schirmer test, and the IgA presence was assessed by a commercial semi-quantitative ELISA allowing the detection of the IgA directed against the main surface antigen of the virus, the S1 protein. Prior to use the assay, the protocol was optimized and validated for IgA detection in tear samples.

The collected results evidenced the presence of a clearly recognizable mucosal response against the SARS-CoV-2 virus in the eye of COVID-19 patients. In fact, ocular anti-SARS-CoV-2 IgAs were detected in 10/28 (35.7%) of the enrolled COVID-19 patients, whereas only one control subject belonging to the sanitary staff subgroup exhibited a weak positivity for the presence of ocular IgA. Since that subject was negative to both SARS-CoV-2 serology and NPS/OPS, the weak presence of ocular anti-SARS-CoV-2 IgA may suggest a possible exposure to the virus in the hospital setting, in the absence of further infection development.

Interestingly, the presence and amount of ocular IgA were seemingly unrelated to the epidemiological features of patients (age, gender), as well as to the lymphocyte number, the presence of ocular symptoms, or the COVID-19 disease stage. High IgA positivity was in fact observed as early as 2 days after NPS/OPS until 48 days after COVID-19 diagnosis and hospitalization Analogously, no correlation was observed between IgA presence/concentration and disease severity, with high IgA amount in both mildly symptomatic (fever, asthenia) and pneumonia patients. However, pneumonia/ARDS was present in only 3/10 (30%) subjects, whereas the remaining patients displayed mild or no respiratory symptoms, possibly suggesting a protective role of mucosal IgA against the progression toward severe symptomatology. The presence of severe symptoms in five cases of IgA-negative patients is suggestive of a possible IgA role in preventing worst outcomes of COVID-19 disease, and the systematic analysis of a larger number of SARS-CoV-2 positive subjects, both asymptomatic and severely ill, may help to clarify this hypothesis. Interestingly, studies on non- human primates showed that animals infected through the conjunctiva had higher viral load in the nasolacrimal system but mild interstitial pneumonia compared to intratracheally-infected animals [39], supporting a protective role of the development of local immunity in the eye.

On the other hand, the lack of detectable IgAs in >50% of our study population might also be correlated to constitutive deficiencies of the immune response (present in six subjects), insufficient tear withdrawal (two patients presented keratoconjunctivitis sicca), or previous prolonged treatment with corticosteroids due to the presence of asthma and allergic rhinitis (in two patients). The enrollment of a larger study population, especially asymptomatic young adults, could indicate whether the mucosal IgA response is developed in most subjects or not.

Notably, the detection of ocular IgA in patients up to 48 days after the first positive NPS/OPS seems to suggest a long persistence of anti-SARS-CoV-2 IgA at the eye level and thus use of IgA detection as a potential diagnostic marker.

Limitations of the present study include the relatively low number of enrolled subjects and the lack of simultaneous serological analyses, due to the unavailability of patients’ blood samples.

However, the presence of a specific anti-SARS-CoV-2 IgA response in the eye strongly suggests deepening the investigation on the specific ocular immunity against the virus, which has been currently poorly explored. Since the virus is infrequently detected in the eye [32,40], the IgA response may indicate that virus antigen presentation occurs in the blood at the lacrimal gland and/or the conjunctival associated *lamina propria* tissue (CALT) [41], rather than by air droplets at the conjunctival sac. This hypothesis should deserve investigation in future studies, to better understand the mechanisms involved in the induction of ocular IgA. Besides, it would be important to assess the impact of IgA presence on the disease course, as the eye displays several defense mechanisms. In this setting, tear IgAs have proven to provide an effective defense against previously isolated SARS-CoV viruses [42].

If confirmed in a larger population, including symptom-free subjects or those with mild symptoms and different times of post-infection, the ocular IgA test could thus represent not only a manageable tool to evidence SARS-CoV-2 infection, which may be extremely helpful in this epidemiological context but also a way to dissect important aspects of the SARS-CoV-2 pathogenesis.

## 5. Conclusions

The pandemic SARS-CoV-2 virus has been reportedly recognized to enter also via the eye surface, and many studies have tried to search and isolate the infectious virus in the eye. Although the presence of SARS-CoV-2 RNA and the clinical presentation of eye symptoms is well documented, the presence of a specific immune response in the eye has been poorly investigated, although this may be very important to better understand the virus pathogenesis.

Our preliminary results, obtained in 28 COVID-19 patients, showed the clear presence of mucosal IgA in the tears of COVID-19 patients with different degree of symptoms and disease stage, suggesting that deepening the investigation on IgA role in the disease may open new perspectives for SARS-CoV-2 patient treatment and that IgA detection may be used as a potential diagnostic/prognostic marker. Furthermore, the systematic search for mucosal IgAs may be crucial to understand how immunity develops in the human host post-exposure to the virus, toward the evaluation of the success of potential vaccine programs.

Meanwhile, waiting for a vaccine, current CDC guidelines rightly recommend protection of the eyes in addition to the nose and mouth, because they contain sensitive mucous membranes, based on the experience of previous SARS/MERS outbreaks and on the presence of a specific mucosal immune response.

## Figures and Tables

**Table 1:** Clinical characteristics and ocular IgA detection in recruited COVID-19 patients.

No	Age	Gender	COVID-19 Symptoms	Ocular Symptoms	Blood Lymphocytes/µL	PCR (mL/L)	Time *	IgA ^§^
1	66	F	Fever, epigastralgia	None	370	0.19	6	1.076 ± 0.02
2	89	F	Fever, dyspnea	None	1010	1.04	47	2.618 ± 0.05
3	73	F	Pneumonia	None	470	3.17	47	9.822 ± 0.13
4	74	F	General malaise, hyporexia, nausea	None	31,430	7.45	43	
5	44	F	Pharyngodynia, anosmia	Mild redness	2290	0.09	54	
6	77	F	Bowel obstruction	None	1690	11.25	11	
7	87	M	Pneumonia	None	1080	2.98	78	
8	80	M	Dyspnea, edema	None	710	4.19	48	1.784 ± 0.03
9	69	F	Cardiac failure	None	1280	3.87	46	
10	59	F	Fever, dyspnea, cough	None	990	3.58	5	0.896 ± 0.01
11	72	F	Gastric hemorrhage	None	1680	0.20	10	
12	58	M	Dyspnea, cough, chest tightness	None	1620	0.83	8	
13	63	M	Fever, pneumonia	None	750	1.31	4	
14	72	M	Heart failure, pleural effusion	None	850	0.60	6	
15	59	F	Fever, asthenia	None	1520	3.76	6	9.402 ± 0.26
16	89	M	Respiratory failure, pneumonia	None	1870	2.44	6	
17	84	M	Acute respiratory failure, pneumonia	None	1540	5.80	3	
18	72	M	Fever, cough	None	1130	14.26	7	
19	62	M	Dyspnea, pneumonia	None	690	1.40	4	
20	75	M	Fever	None	900	3.27	3	2.588 ± 0.51
21	21	F	Pyelonephritis	None	1080	20.21	7	
22	19	F	Fever	None	1670	8.04	2	1.444 ± 0.11
23	72	M	Fever, dyspnea, dysgeusia	None	690	8.31	1	
24	47	M	Recurrent COVID19 recovery	None	1780	0.30	2	
25	68	M	ARDS (acute respiratory distress syndrome)	None	630	28.35	15	2.186 ± 0.09
26	81	F	Fever, dyspnea	None	310	0.75	7	
27	87	F	Fever, bronchopneumonia	None	1710	16.68	7	
28	53	M	Pneumonia, dyspnea	None	1220	0.87	5	3.728 ± 0.23

* Time elapsed between the first positive nasopharyngeal/oropharyngeal swabs (NPS/OPS) and the Schirmer test (days). ^§^ Following the manufacturer’s instructions, positivity was expressed as the ratio ± SD between the absorbance values of samples and calibrator, at OD = 450 nm, expressed as R value as indicated by the manufacturer’s instructions: R < 0.8, negative; 0.8 ≤ R < 1.1, weakly positive; R ≥ 1.1, strongly positive. Each sample was assessed in triplicate.

**Table 2:** Correlation between clinical parameters and ocular IgA detection in recruited COVID-19 patients.

Parameter	Correlation (r) *	95% C.I.	*p* Value
Age	−0.097	−0.46–0.29	0.31 (n.s.)
Gender	0.017	−0.37–0.39	0.46 (n.s.)
Lymphocyte number	−0.199	−054–0.19	0.15 (n.s.)
Ocular symptoms	−0.107	−0.47–0.28	0.29 (n.s.)
Disease severity	−0.133	−0.48–0.27	0.26 (n.s.)
PCR	−0.111	−0.47–0.28	0.29 (n.s.)
Time	0.083	−0.31–0.45	0.34 (n.s.)

* Correlations *r* factor, 95% Confidence Interval (C.I.) and *p* value were calculated by non-parametric Spearman test; n.s., not significant.
